# Using Heartfulness Meditation and Brainwave Entrainment to Improve Teenage Mental Wellbeing

**DOI:** 10.3389/fpsyg.2021.742892

**Published:** 2021-10-15

**Authors:** Ghazal Suhani Yadav, Francisco José Cidral-Filho, Ranjani B. Iyer

**Affiliations:** ^1^Independence High School, Frisco, TX, United States; ^2^Experimental Neuroscience Laboratory (LaNEx), Postgraduate Program in Health Sciences, University of Southern Santa Catarina, Palhoça, Brazil; ^3^Heartfulness Program for Schools, Heartfulness Institute, Novi, MI, United States

**Keywords:** mental health, heartfulness meditation, audio brainwave entrainment, teenager, adolescence, wellbeing, brain training

## Abstract

Teenagers are highly susceptible to mental health issues and this problem has been exacerbated by the quarantine restrictions of COVID-19. This study evaluated the use of Heartfulness Meditation and Audio Brainwave Entrainment to help teenagers cope with mental health issues. It used 30-min Heartfulness meditation and 15-min brainwave entrainment sessions with binaural beats and isochronic tones three times a week for 4 weeks. Using a pretest-posttest methodology, participants were asked to complete a survey battery including the Pittsburgh Quality of Sleep Index, Perceived Stress Scale, Patient Health Question-9, Profile of Mood States, and Cambridge Brain Health assessment. Participants (*n* = 40) were divided into four experimental groups: the control group (*n* = 9), Audio Brainwave Entrainment group (*n* = 9), Heartfulness Meditation group (*n* = 10), and a combined group (*n* = 12), for a 4-week intervention. Data were analyzed with paired *t*-tests. The singular Audio Brainwave Entrainment group did not see statistically significant improvements, nor did any of the intervention groups for brain health (*p* > 0.05). This study, however, proved the efficacy of a 4-week Heartfulness Meditation program to regulate overall mood (*p* = 0.00132), stress levels (*p* = 0.0089), state depression (POMS; *p* = 0.0037), and anger (*p* = 0.002). Results also suggest adding Audio Brainwave Entrainment to Heartfulness Meditation may improve sleep quality (*p* = 0.0377) and stress levels (*p* = 0.00016).

## Introduction

Adolescent depression in the United States is rapidly escalating. From 2007 to 2017, there was a 5% increase in teens aged 12–17 that experienced at least one major depressive episode in the past year (NSDUH, 2017). This rise in depression in the younger demographic affects adolescent development, causing high academic dropout rates, a decrease in social interactions, and high suicide rates (Copeland et al., 2014). Moreover, the unique situation of quarantine owing to the COVID-19 pandemic carries major implications for the mental soundness of teenagers. Research shows that quarantined adolescents are more likely to be “anxious, angry, restless and withdrawn,” suffer from social anxiety and depression, and five times more likely to require mental health services, (Imran et al., 2020; Loades et al., 2020). This is especially exacerbated in this age demographic due to their reliance on peer groups for identity and social support which they lacked due to an absence of social interaction during COVID-19 (Loades et al., 2020). Consequently, this period of self-isolation may amplify the preexisting levels of emotional turbulence that is already prevalent in the young adult population.

Ergo, it has become pivotal to equip teenagers with safer ways to deal with these issues. A highly regarded coping mechanism has been meditation, which endeavors to restrain the tendencies of the mind and bring it to a state of perfect calmness (Balasubramaniam, n.d.; Ross, 2016). More recently, the Heartfulness system of meditation has emerged as a popular meditative practice across the world, with over two million active practitioners globally in 2018 (Heartfulness Institute, 2018). Heartfulness meditation (hereinafter known simply as Heartfulness) is a heart-based practice that aims to attain a balanced mind (Thimmapuram et al., 2017).

Furthermore, audio brainwave entrainment (hereinafter known as ABE), using binaural beats and isochronic tones training, has also become a prominent relaxation technique. Brainwave entrainment refers to the use of rhythmic stimuli to produce a frequency-following response in brainwaves to match the stimuli’s frequency (Huang and Charyton, 2008). For this study, brainwave entrainment audio sessions were provided by the ABE company BrainTap. This study hoped to validate practices such as Heartfulness and the use of ABE as safe and effective means to improve teenage mental wellbeing.

The use of auditory stimuli to induce specific brain states, more specifically, its applications for a variety of mental wellbeing measures, has been a vastly explored topic in academic literature. A study conducted on a senior citizen population by Tang et al. (2015) found that 30 min of daily audiovisual stimulation for a month was seen to improve sleep quality (PSQI), insomnia, and depression. A more time-intensive study on a similar age demographic to that of our study involved 8 weeks of auditory stimulation for adolescent soccer players, also finding positive changes in sleep quality (Abeln et al., 2014). On the other hand, when looking at ABE to improve cognitive functioning, a study by Reedijk et al. saw enhanced attentional control in young adults after simply 6 min of binaural beat listening (2015).

When pertaining to mood states, however, ABE yields contradictory results. A study by Wahbeh et al. (2007a) when using low-frequency delta waves, saw decreases in total mood disturbance, tension, anxiety, confusion, and fatigue subscores of the Profile of Mood States Questionnaire but an increase in the depression and vigor subscores. A later study by Wahbeh et al. (2007b) found the same when using theta waves instead of aforementioned delta waves: in the interventional group, while total mood disturbance decreased, depression subscores increased. Similarly, a paper by Lane et al. also studied varying ABE frequencies. They found an increase in depression subscores when using a lower ABE frequency, but a decrease in the same when using a higher ABE frequency (1998). Thus, not only can it be concluded that the specific frequency of ABE presented in studies is a determining factor for its impact on their participants, but that some frequencies may prove harmful as well. In totality, while larger-scale research on ABE is scarce, studies on the using ABE to ameliorate multiple aspects of mental wellbeing have found mostly positive results.

Heartfulness meditation has garnered the attention of the scientific community, resulting in several studies on the applications of this system. Amarnath et al. (2018) examined how Heartfulness influenced stress and burnout levels of healthcare students. They saw a “mean decrease in workload, worries, tension, and harassment scores and a mean increase in the joy scores” affirming Heartfulness as a mental and emotional support tool. The most significant assertion of this paper, however, was that meditation naturally induces and boosts “Alpha, Theta and Delta” brainwaves (Amarnath et al., 2018). These are the same waves that are artificially induced through audio brainwave entrainment.

A similar study by Mishra et al. (2017) examined the use of Heartfulness for reducing stress in hypertensive patients. Results showed a significant decrease in perceived stress and serum cortisol levels as well as an increase in cognitive functioning. Similarly, Heartfulness was also found beneficial to improve sleep quality and overall quality of life in patients with type 2 diabetes (Sai et al., 2020). Examining papers with a similar age demographic to the proposed study, an investigation by Iyer and Iyer (2019) explored the use of a specific Heartfulness program administered in middle schools. They found an improvement in mental wellbeing (Warwick–Edinburgh Scale) and stress levels (PSS) (Iyer and Iyer, 2019). Thus, there seems to be a consensus on the applicability of Heartfulness in multiple avenues.

As shown, research on both Heartfulness and ABE tends to be concentrated on the adult population, barring the occasional study on adolescents. Their findings, however, cannot be generalized to the teenage demographic because their brain environment starkly differs from the adult brain. Researchers from UC Davis found that the brainwave activity of adolescents differed from that of adults, mentioning a 25% decrease in delta-wave intensity in ages 12–14 and citing another study that discovered similar findings in ages 10–20. This they attributed to reorganization of the brain that occurs during adolescence through synaptic pruning (Feinberg et al., 2006). Furthermore, the brain continues to develop into its adult form well into one’s early twenties, thus making it difficult to extrapolate the findings of previous studies onto the teenage demographic (AACAP, 2016). Moreover, the importance of conducting similar research on adolescents is clearly apparent given the social situation that is unique to them, as explored in the introduction. Additionally, while previous studies on adults seem to arrive at a consensus on the benefits of Heartfulness, the impact of ABE is yet to be clarified. Finally, although there is research to support that brainwaves are induced by meditation, there is no research exploring the use of ABE and artificially-induced relaxation to improve Heartfulness’ results. Regardless of the COVID-19 pandemic, since both Heartfulness and ABE tools are used virtually, they can be used by teens of their own volition, providing them with the ability to take charge of their mental wellbeing without constraints.

Therefore, this study examined the impact of Heartfulness and ABE on the mental wellbeing of high school students through experimental research. We hoped to validate Heartfulness and ABE as useful coping mechanisms for adolescents by exploring their individual effects and how these differed when used in conjunction. Given the documented experimental results obtained from existing research on Heartfulness and ABE, this pilot study hypothesized that Heartfulness may benefit Quality of Sleep, Stress, Mood, and Brain Health. Investigators also hypothesized that using Heartfulness and ABE together may boost the benefits of Heartfulness.

## Methods

### Pre-inquiry Process

#### Study Design

The study made the use of a pretest-posttest experimental design and was conducted in a virtual format.

#### Informed Consent Process

All applicable ethical considerations were made. The study design and inquiry were vetted by the Frisco ISD IRB. Consent was electronically obtained from all participants before the study (in the case of minors, this was provided by parents). Confidentiality was promised to participants as data would be used for further studies after being stripped of identifiable information.

#### Study Subjects

The inclusion criteria for this study were that the participants (1) were high school students in Texas, and (2) had no previous history of mental/neurological illnesses. Participants were enrolled through convenience sampling: contacted through social media platforms and word-of-mouth. A total of 40 participants were divided into four intervention groups using a random name generator. The groups were as follows: the Audio Brainwave Entrainment group (ABE group), Heartfulness Meditation group (Heartfulness group), Meditation and Audio Entrainment group (Combination group), and control group, with 9, 10, 12, and nine participants, respectively. These interventions are discussed in section “Analysis Procedure”. Subjects were awarded volunteer hours for their participation.

### Instrumentation

The questionnaires in the survey battery for the pretest-posttest evaluation have been highly validated as measures of psychometric properties. The variables examined through the study—namely depression, stress, quality of sleep, mood states, and brain health—were chosen to provide a multifaceted profile of each participant’s mental wellbeing.

The Patient Health Questionnaire (PHQ-9) was used to measure subjects’ depression levels through its 9-criteria evaluation; The Perceived Stress Scale (PSS) was utilized to assess the severity of stress; The Pittsburgh Quality of Sleep Index (PQSI) was used to measure participants’ sleep quality; and The Profile of Mood State (POMS) questionnaire was used to assess participants’ mood disturbances, providing participant subscores for anger, confusion, depression, fatigue, tension, and vigor, along with a total mood disturbance score (Buysse et al., 1989).

Finally, the Cambridge Brain Sciences Brain Health Assessment Battery (CBS) was used to measure the brain health of participants. The CBS Standard 4 Tests evaluation consists of four benchmarking tests administered on the CBS platform that measure episodic memory, visuospatial processing, verbal short-term memory, and attention. Results are then compared to those of other participants with similar age and situation from a database of over 10 million tests to provide the patient’s brain performance percentile (Cambridge Brain Sciences [CBS], 2021). This wide availability of comparative tests allows for an accurate yet non-intrusive measurement of participants’ brain health. This tool has been designed and validated by the United Kingdom Medical Research Council: Cognition and Brain Sciences Unit (Owen et al., 2010).

### Intervention

The 4-week-long interventions for each group are as follows:

The ABE group was asked to listen to 15 min of “Intro to BrainTapping” audio—provided through the BrainTap technologies app—three times a week. The session consists of Binaural Beats and Isochronic Tones oscillating (in a sweep mode that changes 1 HZ every 10 s) to produce the following frequencies: 13–30 HZ in the first 2 min; 8–12 HZ from minute 3–12; and 13–30 Hz from minute 12–15.

Secondly, the Heartfulness group was asked to meditate three times a week for 30 min. The participants were introduced to the meditation technique in an introductory group video call by a Heartfulness certified trainer. After this, one of three weekly meditation sessions were held on a group video call led by the principal investigator. These sessions included the Heartfulness Education curriculum that sought to equip the participants with mental health tools, such as affirmations, relaxation, etc. Group sessions served to motivate participants to meditate through peer encouragement.

Thirdly, the participants in the Combination group were asked to complete the tasks pertaining to both the individual interventions.

Finally, the control group was simply asked to complete pretest-posttest evaluation surveys. Follow-up emails were sent weekly to participants throughout the duration of the study.

### Analysis Procedure

Results are presented as mean plus standard error of the mean (SEM). Data were first analyzed for outliers (ROUT test; *Q* = 1%). Next, normality of data was assessed through Anderson–Darling, D’Agostino and Pearson, Shapiro–Wilk, and Kolmogorov–Smirnov tests. The baseline/pretest scores for each group were examined by two-way ANOVA followed by Bonferroni comparisons *post hoc* test. *p* < 0.05 was considered as statistically significant in this analysis. Additionally, pre-post intervention intra-group analysis was conducted using paired *t*-tests. *p* < 0.0125 was considered as indicative of statistical significance. Lastly, mean change score for each intervention was assessed by One way ANOVA followed by Tukey’s multiple comparisons test *post hoc* analysis *p* < 0.05 was considered as indicative of statistical significance in this analysis. The Graph-Pad software (San Diego, CA, United States) was used in all assessments.

## Results

### Participant Demographics

A total of 40 participants (Control = 9, ABE = 9, Heartfulness = 10, Combination = 12) were enrolled in the study. While the participant age group was 14–18, all groups had an average age of ≈16 years. The gender demographic was primarily female, with only an 11% male control group, 22% male ABE group, 30% male Heartfulness group, and 8.3% male Combination group.

### PSS, PQSI, PHQ-9, and Total POMS Results

**FIGURE 1:**
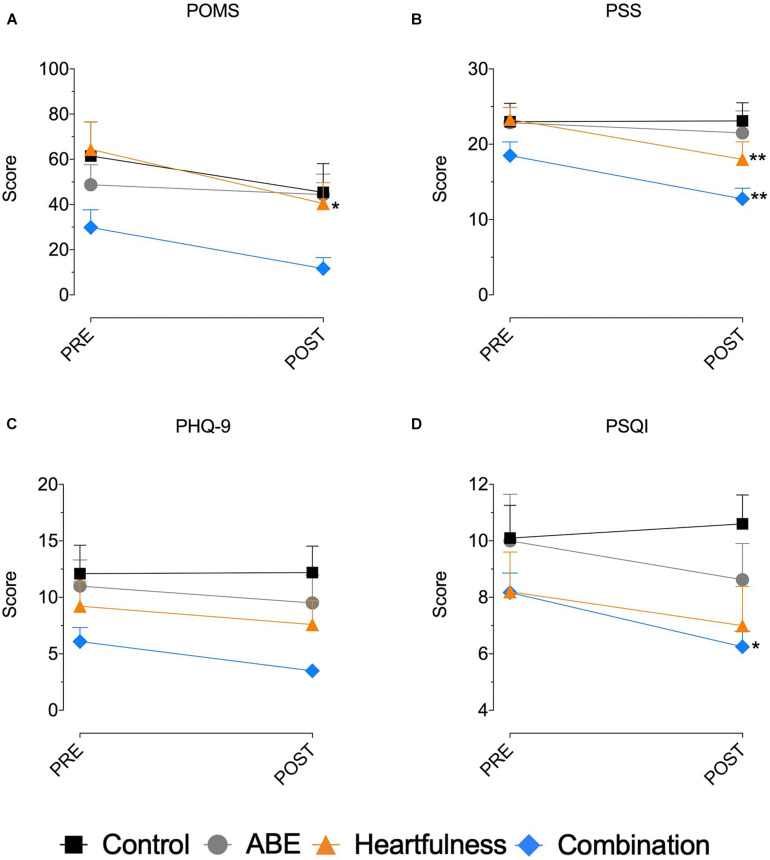
PSS, PSQI, PHQ-9, and total POMS scores. **(A)** POMS, **(B)** PSS, **(C)** PHQ-9, and **(D)** PSQI. *p < 0.05; **p < 0.01.

There were no statistically significant differences between the groups’ baseline evaluations, except for the POMS depression subscores in the Heartfulness group. No outliers were found (ROUT test—*Q* = 1%) and data indicated normal distribution. As shown in Table 1, for all variables analyzed, the ABE group showed no statistically significant improvements. The only statistically significant changes were observed in the Heartfulness and Combination groups. After 4 weeks of intervention, the Heartfulness group (*n* = 10) saw a statistically significant decrease in POMS (*t* = 3.148, *p* = 0.0132) and PSS (*t* = 3.292, *p* = 0.0089). Similarly, for the Combination group, there was a statistically significant decrease in PQSI (*t* = 2.743, *p* = 0.0377) and PSS (*t* = 3.912, *p* = 0.0016) (Figure 1).

**TABLE 1:** Data at a glance.

	**Control (*n* = 9)**			**ABE (*n* = 9)**			**Heartfulness (*n* = 10)**			**Combination (*n* = 12)**		
**Variable**	**Pre**	**Post**	***P***	**Pre**	**Post**	***P***	**Pre**	**Post**	***P***	**Pre**	**Post**	***P***
**Cambridge Brain Sciences**

Episodic memory	99.4	97.0	0.999	96.0	98.5	0.999	102	102	0.999	103	105	0.803
Visuospatial processing	96.8	103	0.106	104	101	0.999	102	102	0.999	101	103	0.999
Verbal short-term memory	101	98.5	0.663	102	101	0.999	99.4	100	0.999	101	102	0.999
Attention	97.8	96.2	0.999	101	102	0.999	97	99	0.999	97	101	0.193
PSS	23.0	23.1	0.999	22.9	21.5	0.999	23.3	18.0	0.009*	18.5	12.8	0.002*
PSQI	10.1	10.6	0.999	10.0	8.25	0.467	8.20	7.00	0.503	8.17	6.25	0.038*
PHQ-9	12.1	12.2	0.999	11.0	9.50	0.999	9.22	7.60	0.188	6.08	3.50	0.107

**POMS**

Total POMS	61.5	45.4	0.164	48.8	44.4	0.999	64.4	40.5	0.013*	29.8	11.7	0.051
Anger	9.70	7.80	0.999	9.13	9.38	0.999	12.7	7.10	0.002*	7.42	3.83	0.172
Confusion	13.7	10.2	0.086	10.1	7.88	0.702	12.0	7.44	0.056	8.58	5.67	0.139
Depression	19.3	15.6	0.445	17.9	14.3	0.644	22.2	14.0	0.004*	5.91	5.00	0.999
Fatigue	11.2	9.70	0.999	9.63	8.00	0.999	12.6	9.00	0.232	7.83	4.33	0.177
Tension	17.4	13.2	0.341	14.6	15.0	0.999	14.3	12.8	0.999	10.7	6.25	0.196
Vigor	9.80	11.1	0.999	12.6	10.1	0.682	9.40	11.3	0.970	12.6	13.4	0.999

*The asterisk refers to the values that are statistically significant.

In the *t*-test analysis, the Heartfulness group saw a significant improvement in total mood disturbance (POMS; *t* = 3.590, *p* = 0.006). The Combination group saw improvements in total mood disturbance (POMS; *t* = 3.263, *p* = 0.008) and stress (PSS; *t* = 5.063, *p* < 0.001).

### POMS Subscore Results

**FIGURE 2:**
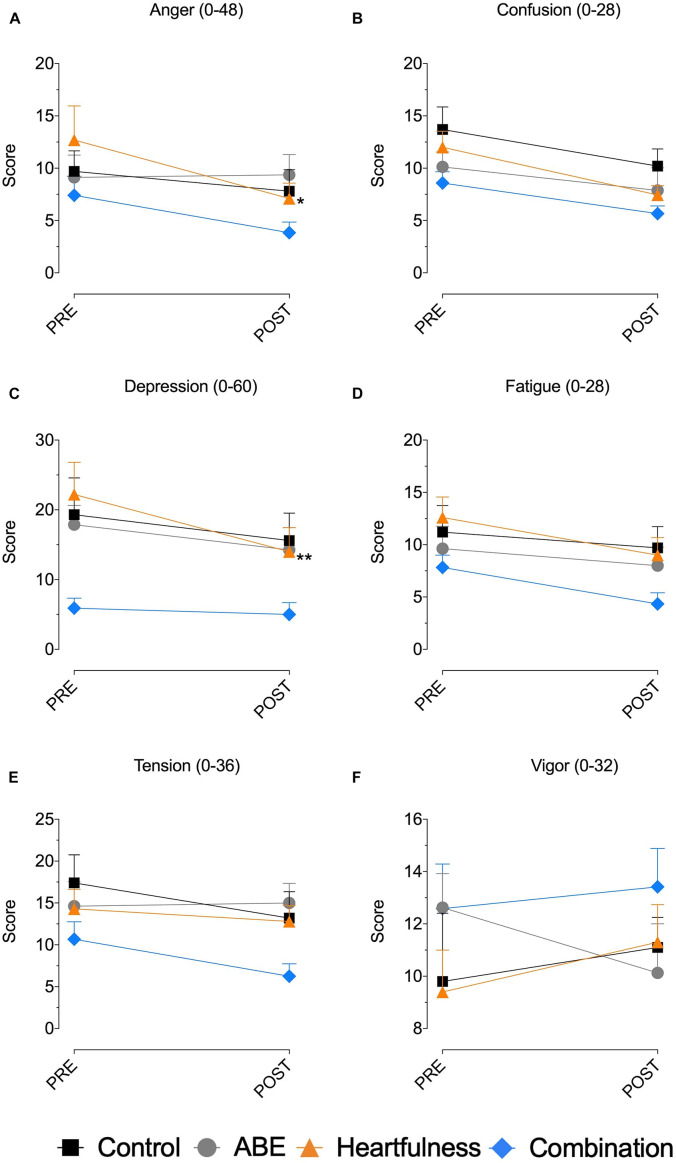
Profile of mood states subscores. **(A)** Anger, **(B)** confusion, **(C)** depression, **(D)** fatigue, **(E)** tension, and **(F)** vigor. *p < 0.05; **p < 0.01.

When examining the subscores of the Profile of Mood States questionnaire, the results were found to be like the aforementioned set of results: the ABE group showed no statistically significant changes in any of the attributes, but there were some significant differences in the Heartfulness groups. As shown in Figure 2, the Heartfulness group witnessed a statistically significant decrease in Anger (*t* = 2.991, *p* = 0.02) and Depression (*t* = 3.621, *p* = 0.0037). The Combination group did not see any significant changes in any of the POMS subscores.

In the secondary *t*-test analysis, Heartfulness group saw a statistically significant improvement in Depression (*t* = 4.259, *p* = 0.002). The Combination group saw decreases in the Confusion (*t* = 3.241, *p* = 0.008) subscores.

### Cambridge Brain Sciences Brain Health Evaluation Results

None of the changes in attributes that were used to measure brain health, including Attention, Episodic Memory, Visuospatial Processing, and Verbal Short-Term Memory, were found to be statistically significant.

### Comparing Efficacy of Heartfulness vs. Combination Interventions

Now, the interventions were compared to prove the hypothesis that the combination intervention was better than the individual interventions. The Heartfulness group saw a statistically significant change in four out of 14 variables, as opposed to the ABE group, which saw changes in none. The Combination group saw a statistically significant change in two out of 14 variables.

Thus, only the Heartfulness group was compared to the Combination intervention. Attributes for which both interventions saw statistically significant results were compared using the mean difference between their pretest and posttest scores. For the primary ANOVA analysis, this meant the PSS score. For the secondary *t*-test analysis, this meant the POMS score. The Combination intervention group saw a higher percentage improvement for both scores: Total POMS score saw a 60.74% decrease and PSS score saw a 30.81% decrease, as compared to the 37.11 and 22.75 decrease in the Heartfulness intervention group. This comparison process is further explained in Table 2. The one-way ANOVA with *post hoc* Tukey’s multiple comparisons test found no statistically significant differences in the changes between the groups, however.

**TABLE 2:** Comparing the combined intervention to the individual intervention.

**Group**	**Heartfulness**			**Combination**			**Comparison**
**Variable**	**Pre**	**Post**	**Percent change**	**Pre**	**Post**	**Percent change**	**Combination > Heartfulness?**
Total POMS	64.4	40.5	37.11%	29.8	11.7	60.74%	Yes
PSS	23.3	18	22.75%	18.5	12.8	30.81%	Yes

## Discussion

This study proved the efficacy of a 4-week Heartfulness Meditation program to regulate overall mood, stress levels, state depression (POMS), and anger. Secondary analysis affirmed the use of the Heartfulness intervention to ameliorate overall mood and state depression (POMS). These findings validate the results of the previously existing although scant body of literature on the same. A previous study by Iyer et al. (2021) found that a 21-day Heartfulness Meditation program administered through a mental wellness app found a statistically significant increase in emotional wellbeing (EPOCH) and a statistically significant decrease in stress levels (PSS). Another study examined the use of Heartfulness meditation with similar measures but in a middle school demographic. They also found an improvement in mental wellbeing (Warwick–Edinburgh Mental Well-being Scale) and stress levels (PSS) (Iyer and Iyer, 2019). Results from both studies are consistent with the findings of our study. All three demonstrate Heartfulness as an alleviating factor for stress levels and wellbeing, albeit through different measures for the latter.

This study also verified adding ABE to Heartfulness to improve sleep quality and stress levels. *T*-test analysis confirmed the utility of the combined intervention to improve overall mood, stress levels, and confusion. Moreover, as hypothesized, this amalgamated intervention saw a greater change in the attributes of overall mood and stress levels, yet the one-way ANOVA and *post hoc* Tukey’s multiple comparisons test did not find these changes statistically significant. This suggests that including ABE with the single 4-week Heartfulness intervention could in fact boost its potency, yet more research is required to prove its statistical significance.

Perhaps, the artificial inducement of brainwaves through the entrainment audio enhanced the relaxed states attained during meditation. This could be verified through further research exploring the interconnection of the interventions at a more neuroscientific level. The lack of literature combining both interventions, however, prevents us from comparing these findings with that of previous studies.

While the singular ABE intervention did not yield significant results, this could possibly be attributed to the disparity in the time duration of the interventions; the ABE intervention entailed 15 min of audio listening while the Heartfulness groups were asked to complete 30 min of meditation. This reduced listening duration could also be the reason why the results of this study varied from the largely positive findings of other studies. Both this study and Abeln et al. (2014) used theta, alpha, and beta frequencies in the auditory intervention, conducting the study on a largely similar age demographic. Abeln et al. (2014), however, played their audio throughout the participant’s sleep for 8 weeks, causing a significantly large difference in intervention time between their study and ours (Abeln et al., 2014). This could be the reason for the contrast between their results and ours, wherein they found improvements in the PQSI’s sleep quality measure, but ours were statistically insignificant. On the other hand, Reedijk et al. (2015) simply used 6 min of audio entrainment: three before and three during intervention. They saw that the gamma frequency intervention caused the elimination of the attentional blink phenomenon in participants (Reedijk et al., 2015). Perhaps, in this case, the difference in duration of intervention was not the cause of the discrepancy between their positive results in terms of attentional control and our non-results in the same domain; it could be attributed to the difference in frequency of audio that was used. As mentioned in the introduction, comparing studies like Lane et al. (1998) and Wahbeh et al. (2007b) makes it clear that the frequency of audio can have a polarizing effect: while this study did not find a negative impact on the mood of participants when low-frequency audio was used, Wahbeh et al. (2007b) did. Thus, the findings of this paper further perpetuate the preexisting disaccord in the academic community regarding the effectiveness of audio brainwave entrainment.

Limitations of this pilot study include the (1) shorter duration, (2) female-skewed volunteer distribution, and (3) small convenience sample. These create a need for more extensive and longitudinal studies to consider our findings indisputable. Moreover, the questionnaires used were numerous, potentially causing fatigue in participants while filling them out; this could cause their real mental states to be inconsistent with what was submitted. Additionally, due to the virtual nature of the research study, there was no means to ensure that all sections of the intervention were followed and completed within a timely manner. Results were analyzed solely on the assumption that the participants had completed their intervention. Finally, despite the randomization of the groups, the baseline differences for the POMS depression subscores were statistically significant.

The results of this study, however, warrant further longitudinal, cross-sectional research that consider the limitations of this study and corroborate these findings. In accordance with these limitations, it would be beneficial to assess these interventions in an in-person format to ensure the participation of all subjects while examining a larger, more diverse sample size. Additionally, exploring these interventions and their impact on teenage mental wellbeing at a neuroscientific level would help attribute the connections of the study to causation factors instead of mere statistical correlations.

This study addressed the originally identified gap, exploring the application of interventions—previously researched on adult populations—in the adolescent demographic. Due to the current situation of pandemic-associated isolation, the mental health of teenagers is quickly deteriorating. Considering these circumstances, this study’s results are optimistic because they validate specific practices that teenagers can use of their own volition while conforming to social distancing and quarantine guidelines, improving emotional wellbeing while not endangering physical health.

Regardless of such circumstances, the teenage mind has always been vulnerable and “primed” for mental stability issues. The neural restructuring that marks adolescence is also accompanied by susceptibility to mood disorders which are further exacerbated by the increased biological need for sleep in this development stage (Jansen, 2015). This study not only found an improvement in overall mental wellbeing through Heartfulness and Combination interventions, but also saw a specific improvement in the sleep quality of the Combination group. By improving particular vulnerabilities of the adolescent brain, namely, sleep deprivation, the study shows significant applicability to help combat the general imbalances of the developing teenage brain.

The onset of mental health issues is now being observed in an increasingly younger demographic, warranting crucial research into a variety of mental health support tools. However, limited the scope of the study, its claims provide a unique insight into alternative mental support channels for adolescents aged 14–18.

## Data Availability Statement

The datasets presented in this study can be found in online repositories. The names of the repository/repositories and accession number(s) can be found below: Figshare Repository: https://figshare.com/articles/dataset/Using_Heartfulness_Medita tion_and_Brain_Entrainment_to_Improve_Teenage_Mental_W ellbeing/14825682.

## Ethics Statement

The studies involving human participants were reviewed and approved by Frisco ISD Internal Review Board. Written informed consent to participate in this study was provided by the participants’ legal guardian/next of kin.

## Author Contributions

FC-F and GY conceived the experiment and designed it with the help of RI. GY performed the experiment, analyzed the data, and drafted the manuscript. All authors contributed to the article and approved of the final copy.

## Conflict of Interest

FC-F is employed by BrainTap, the company that provided the ABE sessions. RI works for the Heartfulness Institute, but due to the absence of a commercial product from the organization, there is no financial interest. The remaining author declares that the research was conducted in the absence of any commercial or financial relationships that could be construed as a potential conflict of interest.

## Publisher’s Note

All claims expressed in this article are solely those of the authors and do not necessarily represent those of their affiliated organizations, or those of the publisher, the editors and the reviewers. Any product that may be evaluated in this article, or claim that may be made by its manufacturer, is not guaranteed or endorsed by the publisher.
